# New Immunosuppressive Therapies in Uveitis Treatment

**DOI:** 10.3390/ijms160818778

**Published:** 2015-08-11

**Authors:** Salvador Mérida, Elena Palacios, Amparo Navea, Francisco Bosch-Morell

**Affiliations:** 1 Instituto de Ciencias Biomédicas, Universidad CEU Cardenal Herrera, Valencia 46113, Spain; E-Mails: salvador.merida@uch.ceu.es (S.M.); navea_amp@gva.es (A.N.); 2 Oftalmología Médica, Fundación para el Fomento de la Investigación Sanitaria y Biomédica de la Comunitat Valenciana, Valencia 46020, Spain; E-Mail: eleofta@hotmail.com

**Keywords:** uveitis, corticoids, cytokines, antibody, immunosuppressive

## Abstract

Uveitis is an inflammatory process that initially starts in the uvea, but can also affect other adjacent eye structures, and is currently the fourth cause of blindness in developed countries. Corticoids are probably the most widespread treatment, but resorting to other immunosuppressive treatments is a frequent practice. Since the implication of different cytokines in uveitis has been well demonstrated, the majority of recent treatments for this disease include inhibitors or antibodies against these. Nevertheless, adequate treatment for each uveitis type entails a difficult therapeutic decision as no clear recommendations are found in the literature, despite the few protocolized clinical assays and many case-control studies done. This review aims to present, in order, the mechanisms and main indications of the most modern immunosuppressive drugs against cytokines.

## 1. Introduction

The term uveitis encompasses any inflammatory process that affects the uveal tract (iris, ciliary body, and choroids) and its adjacent structures (vitreous humour, retina, optic nerve, and vessels). The origin of this inflammatory process can be of endogenous, by forming part of a systemic disease or mainly an eye syndrome, or exogenous origin, related above all to infectious factors that affect the eyeball in an isolated fashion or take place in multisystemic infections.

Uveitis is, anatomically, classified according to the location of inflammation, which can be anterior (anterior chamber), intermediate (vitreous), posterior (choroidea and retina), and panuveitis (anterior chamber, vitreous, choroidea, and retina). Uveitis may also be classified clinically as infectious (bacteria, viral, fungal, parasitic, *etc.*), noninfectious (known or unknown systemic association), and masquerade (group of eye diseases that mimic chronic intraocular inflammation).

Anterior uveitis is the most frequent form of uveitis (42%–54%) for all populations and ages, especially in the Western world, where prevalence of human leukocyte antigen B27 (HLA-B27) is high (32%). HLA-B27 is clearly a risk factor for developing anterior uveitis, as Brewerton *et al.* described in the 1970s [1]. Studies have shown that prevalence of HLA-B27 is much different in Eastern populations, like Japan, India or the Arabian Peninsula, where it is present in only 6%, 2% and 1.3%, respectively, and anterior uveitis (the most frequent location) is mostly idiopathic (80%) [2].

In the Western world, posterior uveitis and panuveitis are the second and third most frequent locations with 21% and 7%, respectively [3]. These figures increase considerably in South American or African countries (20%–28%) [4], where posterior uveitis predominates, especially for Toxoplasma due to poor health-hygienic conditions [5], and in Asian countries (41%–43%) with a high rate of panuveitis cases secondary to Vogt-Koyangi-Harada and Behçet’s syndromes [6]. Finally and globally, intermediate uveitis is doubtlessly the least frequent location, with an estimated incidence of 1.5–2.08 per 100,000 in Western populations [5] and whose cause is mainly idiopathic. However, it is the second most frequent location in patients under age 16 in whom it can represent up to 28%, depending on the series under study [7].

Comparisons among diverse regions are problematic because of extensive geographic discrepancy in both disease aetiology and clinical features, and the heterogeneity of uveitis entities [5]. Nowadays, the distribution of all the uveitis types in Western countries is changing as migration to better developed places with more opportunities is increasing. It is not surprising to find causal factors of this change in large multi-ethnic urban populations, as more recent cross-sectional studies have observed [8].

In etiology terms, we classify all uveitis types as infectious and non-infectious, and we include secondary uveitis cases in systematic autoimmune diseases or mainly eye syndromes.

From all these facts, we realize how relevant a detailed anamnesis is that reflects not only an eye examination in the clinical history (localition, bilateralism, *etc.*), but also patients’ epidemiological and demographic characteristics, like age, gender, race, toxic habits, systemic symptoms, *etc.* The correct establishment of general data about the patient (anamnesis) may limit diagnosis in terms of the number of causing entities in order to avoid patients undergoing irrelevant complementary tests that have a high cost-benefit; e.g., starting the HLA-B27 test in an oriental citizen with anterior uveitis when we know that prevalence of antigen positivity is very low in oriental populations.

Adequate and comprehensive patient diagnosis has important prognostic and therapeutic implications, allowing recognizing the potentially lethal systemic diseases.We should also attempt to make an etiologic diagnosis of uveitis to avoid iatrogenic effects through unsuitable treatments. It is vitally important to rule out the infectious pathology for which great care must be taken when using corticoids or inmunosuppressors. It is estimated that 74% of uveitis cases can be correctly classified [8]. Once again, distribution varies according to the study population, but 29% of uveitis cases are infectious in Western communities (secondary to Toxoplasm, tuberculosis, and the Herpes Virus family), 25% are associated with immune-mediated systemic diseases (seropositive and seronegative spondyloarthropathies, sarcoidosis, Behçet), 20% are typical eye syndromes (Birdshot chorioretinopathy), and 26% are unclassifiable [5,8].

Although annual uveitis incidence is not high and varies according to the study population, with a range of 17–52 people for every 100,000 inhabitants, its appearance has serious consequences for patients as it appears in the 20–60 year-old age group in 70%–90% of cases. This age group is considered to be of working age. Uveitis considerably affects productivity and quality of life, partly because it is the fourth cause of blindness in developed countries, specifically 10%, which rises to 24% in developing countries [9,10].

Visual prognosis will depend on etiology and uveitis type, but anterior uveitis has better visual prognosis, while that of posterior and panuveitis is worse. Among the causes of irreversible visual loss we find glaucoma and macular lesions, like scars or refractory cystoid macular edema, vascular retinal alterations, retinal detachment, optic nerve atrophy, and phthisis [9,10].

## 2. The Eye’s Immune Privilege

Eyes, along with the brain, placenta, and testicles, have certain immune characteristics which allow them to maintain a low level of immunity and also tissue integrity against undesirable and irreversible effects that can cause an inflammatory response and lead to visual loss. This is known as “immune privilege”. To achieve it, the eye has anatomical mechanisms, like a physical barrier called the hemato-retinal barrier and lack of lymphatic drainage, and also molecular mechanisms, such as secretion of soluble immunosuppressive factors by eye cells, such as β-TGF, Fas ligand (FasL) or the low expression of MHC class II molecules in antigen-presenting cells [11]. These mechanisms also help inactivate the immune response of pigment epithelium cells with similar characteristics to macrophages, which are capable of secreting anti-inflammatory cytokines and Müller cells, and have been shown to inhibit the proliferative capacity of T-cells in cultures [11,12].

Peripheral tolerance is sustained by antigen-specific T regulatory cells (Tregs). Thus the location of alloantigens in the anterior chamber brings about a form of immune tolerance identified as anterior chamber-associated immune deviation (ACAID), which induces antigen-specific CD8^+^ Tregs and contributes to the eye’s immune privilege by down-regulating immune responses. Treg cells avoid autoimmune diseases by upholding self-tolerance and defeating pathogen-induced immunopathology [12].

Uveitis will appear, therefore, if maintaining this perfect immune balance is not possible when faced with aggression of an endogenous or exogenous kind. It is followed by the consequent increase in proinflammatory cytokines (IL-2, IL-12, TNF-α, INF-γ, *etc.*) as opposed to anti-inflammatory ones (IL-4, IL-10, INF-α, *etc.*), chemotactic factors, complement system activation, antigen-presenting cells, T-lymphocytes, B-lymphocytes, endothelial cells, *etc.* [13,14].

## 3. T Helper Cells, Cytokines and Uveitis Treatment

**Figure 1:**
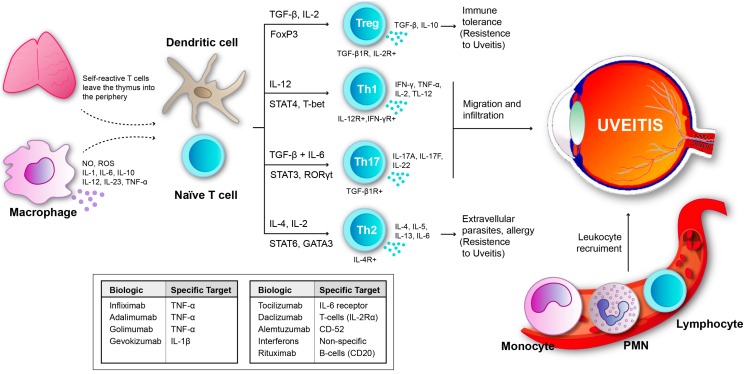
Uveitis results from imbalance between inflammatory mechanisms and regulatory mechanisms. Acute inflammation initiated by those cells that are previously present in affected tissues, mainly resident macrophages and dendritic cells [12]. In autoimmune uveitis, self-reactive T cells leave the thymus and when they reach the eye they come in contact with retinal antigens. Myeloid dendritic cells present a solid ability of capturing antigens, which enables them to stimulate T cells. Therefore, T-lymphocytes may differentiate into Tregs, Th1, Th17 or Th2 for precise immune response in function of the antigen encountered and cytokine presence. Th1 and Th17 cells participate in inflammatory and autoimmune uveitis. Th1 cells are crucial for the development of uveitis, whereas Th17 cells play a relevant role in the late/chronic phase of uveitis, however induced Treg cells defeat both Th1 and Th17 cell responses [13,14,15,16,17]. Furthermore, the migration of Th1 and Th 17 to the eye, also results in the breaking down of the blood-retinal barrier and, consequently, different leukocytes from the circulation are recruited. In the figure, we can also see the main biologics that are being presently used in uveitis therapy and its targets.

Since the 1980s, the T helper cell types 1 and 2 (Th1 and Th2) paradigm has been considered the cornerstone of immune responses. Th1 cells, which secrete IL-2, INF-γ, and lymphotoxin, are critical for macrophage activation and nitric oxide production [12]. Th2 cells, which secrete IL-4, IL-5 and IL-13, mediate for humoural immune responses. However, a third subset of T helper cells, called Th17 cells, was discovered and characterized 10 years ago [15,16]. Thus inflammatory components include Th1 and Th17 lymphocytes that produce pro-inflammatory cytokines such as IL-2, INF-γ, IL-17, IL-23 and TNFα, which recruit leukocytes from circulation to result in tissue damage (Figure 1). Experimental uveitis disease induction has been characterized by the polarization of early T helper (Th) 0 or Th2-like responses to Th1 and Th17, whereas resistance to disease is associated with both regulatory cells and polarisation towards a Th2 pathway [17,18,19].

Determining suitable treatment for each uveitis type involves hard therapeutic decision-making as no clear recommendations are found in the literature, despite the few randomized clinical assays performed and the many case series or case-controls conducted. We must read their results more critically as the information they provide may seem an irrefutable scientific dogma and could, in fact, be based on non-conclusive data obtained from very few patients, and from the four basic clinical studies (case series, case-control studies, cohorts, and randomized assays), which are more ineffectual to obtain relevant clinical data.

## 4. Corticosteroids

The greatest advance in caring and treating patients with immunological-based diseases was made in 1952 when efficient therapy with corticosteroids was discovered. Since then, their use has extended to primary ophthalmological practice first in systemic and topical usages, and more recently with the appearance of new slow-release intraocular devices.

Corticoids form the first-line therapy in uveitis cases. The anti-inflammatory properties of corticosteroids are mediated by cytosolic glucocorticoid receptors which rise expression of anti-inflammatory proteins. These receptors, members of the nuclear receptor family of transcription factors, have been found in the ciliary body, corneoscleral tissue, and iris [20]. It has commonly thought that the anti-inflammatory feature of glucocorticoid receptors results from the transrepression of proinflammatory genes by the binding of the monomeric glucocorticoid receptor to other transcription factors. However some recent studies, which used mutant mice in which the glucocorticoid receptor was mostly dimerization defective due to the replacement of an alanine with a threonine, have shown that glucocorticoid receptor dimers also account for resolving inflammation [21].

Topical corticoids are efficient in anterior uveitis, as Dunne and Travers [22] observed in a controlled double-blind assay when comparing betamethasone with a placebo in patients with non-granulomatous unilateral anterior uveitis. The problem with topical corticoids is that they offer poor activity in the other locations (intermediate, posterior, and panuveitis). Anterior uveitis comprises a heterogeneous group of diseases like the idiopathic kind, associated with HLA B27, juvenile idiopathic arthritis (JIA) associated with Behçet, *etc.* Most cases respond well to topical treatment, along with mydriatic collyriums, and offer an excellent prognosis, provided the correct treatment is administered early. Yet there are some exceptions to the rule, where uveitis may indicate strong activity and tends to require systemic immunosuppression with a topical treatment, as with Behçet or JIA [20].

In serious cases of anterior uveitis, unilateral intermediate uveitis, and as an adjuvant in posterior uveitis, periocular corticoids are effective if administered rapidly and with the anti-inflammatory drug admininistered at a high concentration. Dafflon *et al.* [23] observed after administering periocular steroids that 60% of patients with posterior uveitis gained 2–5 Snellen lines and 19% gained more than five lines. Monitoring intraocular pressure (IOP) in these patients is very important because using this therapy implies increased IOP in a not-inconsiderable percentage of patients (43% in Dafflon’s group).

One of the problems with this form of administration is the wide variability in the intraocular concentrations of corticoids in different patients because of the limited diffusion of steroid particles through Tenon’s capsule and sclerotics. This situation has been overcome with an intravitreal injection of corticoids, as Roesel *et al.* demonstrated [24], and this form is superior to the local form. In 2010 the Food and Drug Administration (FDA) approved a biodegradable slow-release dexamethasone implant for venous thrombosis-related cystoid macular edema. After demonstrating its efficacy and safety in a randomized double-blind study, its use extended to macular edema secondary to non-infectious intermediate or posterior uveitis [25].

The oral administration of corticoids is the initial treatment used in non-infectious posterior uveitis and panuveitis cases. Nussenblatt *et al.* [26] performed the first randomized double-blind assay in 56 uveitis patients, which compared prednisone with cyclosporine, and found no differences in efficacy. The initial prednisone dose which treatment began with was 1 mg/kg/day, and it was continued until a satisfactory anti-inflammatory response was obtained, normally after one month. Next, the dose was gradually reduced, but for no longer than 3–6 months, due to serious systemic side effects like increased body weight, hypertension, diabetes, hypercholesterolaemia, gastritis, gastrointestinal ulcers, premature epiphyses closure in children, retarded growth, osteopenia, osteoporosis, *etc.* [20]. Higher corticoid doses (10 mg/day) have been reported to not only inhibit calcium from diet being absorbed, but to also suppress the response to calcitonin, which increases bone reabsorption. Loss of bone is inevitable during therapies with steroids, and 4%–5% can be lost in the first six months. Effects in the spine and femur tend to be more marked, with a 2.5-fold higher risk of fractures compared with the general population [27]. In 2010, the American College of Rheumatology published some recommendations to prevent and treat corticoid-induced osteoporosis in patients who, regardless of dose, took corticoids for at least three months. This College stressed changing certain patient habits, like drinking less alcohol, stopping smoking, and practicing physical exercise, and associating it with calcium (1200–1500 mg/day) and vitamin D (800–1000 UI/day) intake [28].

If certain undersirable side effects appear with corticoid therapy, or if the disease becomes recurrent after leaving corticoids or does not subside, starting therapy with immunosuppressors is indicated in order to permanently withdraw corticoids.

## 5. T-Lymphocyte Inhibitors

The most widely used immunosuppressors are T-lymphocyte inhibitors, of which cyclosporine A is stressed because its efficacy compares with corticoids [26].

Cyclosporine is produced in nature by diverse fungi, such as *Beauveria nivea*, and is an 11-amino acid cyclic peptide which generates immunosuppressive effects by binding to cyclophilin of T-lymphocytes. In this way, the cyclosporine-cyclophilin complex inhibits calcineurin, which triggers the downstream cascades needed for the transcription of IL-2, CD40 ligand and Fas ligand. Specifically, the cyclosporine-cyclophilin complex inhibits calcineurin phosphatase induced upon T-cell activation by suppressing IL-2 synthesis [29]. Simultaneously Tregs, a subpopulation of T-cells which modulate the immune system and preserve tolerance to self-antigens, resist cyclosporine-induced cell death via CD44-mediated signaling pathways [30].

Nussenblat *et al.* [26] were the first to publish a work on the efficacy of cyclosporine taken at a dose of 10 mg/kg/day in patients with uveitis secondary to several aetiologies: sarcoidosis, Behçet, Birdshot, pars planitis, Vogt-Koyanagi-Harada, *etc.* [31]. However, these authors also observed that taking cyclosporine at this dose was associated with hypertension and nephrotoxic effects in 100% of cases. Thus, the suitable dose, with proven efficacy and fewer adverse effects, is considered to be 2–5 mg/kg/day; at this dose, only 10.7% of patients abandoned treatment because of undersirable effects. The multicentre SITE (the Systemic Immunosuppressive Therapy for Eye Diseases Cohort Study) Study conducted in the USA stated that the benefits of using cyclosporine were modest since it observed that it offered a complete and sustained solution for inflammation in only 33.4% and 51.9% of patients at six months. This study also found that minimum inflammation remained in 25% of patients over the same treatment period. This study also advised not using this drug in people aged over 55, in whom the complications rate rose [32].

## 6. Antimetabolites

Among antimetabolites, we stress methotrexate (MTX), which has been widely used to treat inflammatory eye diseases ever since Wong and Hersh [33] observed its efficacy in the 1960s. MTX is an antimetabolite of folic acid that plays by competitively binding to enzyme dehydrofolate reductase, thereby blocking the formation of purines and pyrimedines within the cell, and consequently inhibiting DNA synthesis. MTX may also delay protein synthesis by impeding the conversion of some amino acids. Hence it inhibits cell growth and proliferation by depleting the pool of reduced folates or tetrahydrofolates, and may also induce T-cell apoptosis, modification of the B-cell response and cytokine production inhibition [34].

It is worth highlighting that its use is based on the data collected from retrospective case series and uncontrolled patients with uveitis associated with JIA, sarcoidosis, pemphigus, rheumatoid arthritis, *etc.* [31] Once again, the SITE study informed that MTX caused eye inflammation to completely subside in 66% of the 384 patients studied in one year. These data suggest that MTX is more beneficial than cyclosporine, which obtained a result of 51.9% during the same period. Even so, MTX was described to have no beneficial effects in 13% of the cases. The rate of complications associated with this drug (myelosuppression, hepatotoxicity) led 16% of patients to drop treatment [35].

Another immunosuppressor drug evaluated by the SITE group was mycophenolate mofetil. After evaluating 236 patients treated in several hospital clinics in the USA, it was observed that the remission rate at six months was 53%, and 73% after one year, this drug was well-tolerated, and only 12% dropped treatment because of side effects [36].

## 7. Biologics

Nowadays, advances in molecular research have evidenced the key role that the deregulation of inflammatory cytokines plays in the pathogeny of immune-mediated diseases, and has allowed new targeted therapies to be developed which interfere with how specific molecules function which, in turn, cause inflammation and tissue damage. They are known as biological therapies and emerge, in principle, when we intend to treat chronic systemic immunological-based diseases in the rheumatology field. Later they were adopted to be used with uveitis, but are now no longer indicated despite their clinical efficacy reported in publications of cases and case series [37]. Thus biologics has been manufactured by recombinant DNA technology and includes different drugs, such as monoclonal antibodies, soluble receptors, cytokines themselves, and natural cytokine antagonists (Table 1).

Many new biologics are being developed, which include inhibitors of IL-17, among others. Although these seem promising, their ultimate utility cannot be currently assessed. We now go on to state those that are being presently used in uveitis therapy.

**Table 1:** Biological therapies to treat chronic systemic immunological-based diseases related to uveitis.

BIOLOGICAL AGENT (FDA Initial Approval Date)	BRAND NAME	MECHANISM OF ACTION	PRINCIPAL INDICATIONS
**Proinflammatory cytokine inhibitors**			
Infliximab (1998)	Remicade	Anti TNF-α	CD, UC, RA, PA, AS, Ps
Etanercept (1998)	Enbrel		RA, PJIA, PA, AS, Ps
Adalimumab (2002)	Humira		RA, PJIA, PA, AS, CD, Ps
Golimumab (2009)	Simponi		RA *, PA, AS
Gevokizumab		ANTI IL-1β	PG **
Tocilizumab (2010)	Actemra	ANTI IL-6R	RA, PJIA
**T-cells inhibitors**			
Daclizumab (1997)	Zenapax	ANTI CD25 (IL-2R)	PRR
**B-cells inhibitors**			
Rituximab (1997)	Rituxan	ANTI CD 20	RA *, CLL, n-HL
Alemtuzumab (2001)	Campath	ANTI CD 52	CLL
**VEGF inhibitors**			
Bevacizumab (2004)	Avastin	ANTI VEGF	MCC

* in combination with methotrexate; ** phase-three program was initiated in November 2014; AS: Ankylosing Spondylitis; CD: Crohn’s Disease; CLL: Chronic Lymphocytic Leukemia; MCC: Metastatic Colorectal Cancer; n-HL: non-Hodgkin’s lymphoma; PA: Psoriatic Arthritis; PG: Pyoderma Gangrenosum; PJIA: Polyarticular Juvenile Idiopathic Arthritis; PRR: Prophylaxis of Renal Rejection; Ps: Psoriasis; RA: Rheumatoid Arthritis; UC: Ulcerative Colitis.

### 7.1. Etanercept

Aqueous humour and sera of patients with uveitis present high TNF-α levels [38]. The first anti-TNF used in assays in humans was Etanercept, a fusion protein of two p75 TNF receptors, and a human Fc molecule which binds free TNF-α and TNF-β. The first assays date back to the beginning of the 1990s [39] and showed a beneficial effect in patients with psoriasis, either associated or not with arthritis, rheumatoid arthritis, or ankylosing spondylitis. Yet no clear efficacy has been shown to date in uveitis patients like Infliximab or Adalimumab has done. Indeed exacerbations in inflammation have even been described in some cases, which have been attributed to the drug itself [40,41].

### 7.2. Infliximab

Infliximab is a chimeric IgG1κ monoclonal antibody composed of human-constant and murine-variable regions, and is specific for human TNF-α. It inhibits the biological activity of TNF-α by both binding with high affinity to the soluble and transmembrane forms of TNF-α and blocking receptor binding. Infliximab was approved by the FDA in 1998 for Crohn’s disease, and later for ulcerative colitis, psoriatic arthritis, rheumatoid arthritis, and ankylosing spondylitis. Its benefit for uveitis secondary to JIA and Behçet is no longer indicated. In a prospective study, Sfikakis *et al.* [42] observed how vitritis, macular edema, and vasculitis were resolved in 28 days in 90% of the 25 patients enrolled with uveitis for Behçet. This therapy has also been described with good results in cases of sarcoidosis, Vogt-Koyonagi-Harada, Birdshot, *etc.*

Several studies conducted with animal models have proved the efficacy and safety of Infliximab in uveitis [43], dry eye [44], or in scarring processes on the eye’s surface [45]. Clinical assays have indicated its rapid beneficial effect after being intravenously administered. However, it has been described to have some significant adverse effects, such as thrombo-embolism, LUPUS-like syndrome, and the formation of solid tumours or lymphomata. In a recent study with 72 patients, Kruh *et al.* [46] found that Infliximab induced a high rate of whole clinical remission in recalcitrant uveitis and was well tolerated by most patients. However, 36.4% of the patients experienced at least one side effect while on infliximab therapy.

As 25% of the molecule is of murine origin, many patients develop antibodies against the drug, which reduces its beneficial effect. However, this effect is reduced when associated with MTX [47].

### 7.3. Adalimumab

Adalimumab is a monoclonal antibody that inhibits factor TNF-α and, unlike Infliximab, it is totally human, and offers the added advantage of being used subcutaneously, and can be self-administered by patients every two weeks. It was approved by the FDA for rheumatoid arthritis in 2002, and its indications were later extended to psoriatic arthritis, Crohn’s disease, ulcerative colitis, ankylosing spondylitis, and juvenile arthritis.

In recent years, its use has extended to childhood refractory uveitis. Shortly after 2002, Adalimumab seemed an effective safe therapy for refractory uveitis management [48]. It might provide the possibility of reducing the use of concomitant immunosuppressive drugs in these patients. In fact, Adalimumab offers numerous advantages over Infliximab; e.g., easier administration, better patient compliance, cost-effectiveness, and fewer adverse cases. Retrospective studies have shown its effectiveness in uveitis secondary to JIA as inflammation subsided in 80% of the cases after six weeks of treatment [49]. Adalimubab seems to be the most effective biological agent in JIA as it is capable of controlling inflammation in 35% of refractary uveitis in children who had not previously responded to Infliximab or Etanercept [50].

In clinical assays, it has been demonstrated as being efficient in uveitis secondary to Behçet, sarcoidosis, Birdshot, Vogt-Koyonagi-Harada, in uveitis refractory to usual treatment [47], and after being used as an adjuvant with other immunosuppressors, where a 50% reduction in the immunosuppressor dose was observed in 85% of patients at six months [51]. Clinical efficacy correlates with both the up-regulation of Tregs and the modulation of VEGF-mediated inflammatory pathways [52].

### 7.4. Golimumab

Golimumab is another human IgG1 anti-TNF-α monoclonal antibody; its high affinity and specificity for human TNF-α effectively neutralizes TNF-α bioactivity *in vitro*. Therefore, the affinity of Golimumab for soluble human TNF-α, as determined by surface plasmon resonance [53], is similar to that of Etanercept (18 *versus* 11 pM), is higher than that of Infliximab (44 pM), and is much higher than that of Adalimumab (127 pM, *p* = 0.018).

Using Golimumab in rheumatoid arthritis, psoriatic arthritis, ulcerative colitis, and ankylosing spondylitis has been approved when used subcutaneously and on a monthly basis. Some publications were found on isolated cases of recalcitrant uveitis secondary to JIA who did not respond to either Infliximab or Adalimumab, but responded to Golimumab two weeks after starting treatment [54,55]. Its efficiency has also been reported every three weeks instead of four weeks [56]. A good response to treatment and follow-up in a patient with uveitis because of spondyloarthropathy [57], or vitritis and macular edema completely remitting after five weeks in a Behçet patient [58], makes us think that Golimumab is a good alternative in refractory patients who do not respond to other anti-TNF-α.

### 7.5. Gevokizumab

IL-1α and IL-1β exert strong proinflammatory effects. An IL-1α intravitreal injection causes intraocular inflammation, which agrees with the IL-1 concept and opens up a cascade of inflammatory mediators [59]. IL-1β results mainly from activated macrophages, but also from vascular endothelial cells and B-cells. An IL-1β intravitreal injection also causes intraocular inflammation in relation to the breakdown in the blood-retinal barrier [60]. Recently, Zhao *et al.* [61] suggested that IL-1β might be an important mediator in the pathogenesis of autoimmune diseases, such as uveitis.

Gevokizumab is a potent monoclonal antibody which binds strongly with IL-1β. Gevokizumab taken as a single 0.3 mg/kg dose has been found to be effective in seven patients with uveitis and severe vasculitis secondary to Behçet, with complete improvement in all patients after two weeks. This effect continued for the 98 days that the study lasted and no related side effects were noted [62].

### 7.6. Tocilizumab

Some studies have found significantly high IL-6 in ocular fluids such as vitreous or aqueous humours, which derives from refractory/chronic uveitis patients and animal models [19,63,64]. However, we found very few articles that referred to using Tocilizumab in uveitis, and those we found were, once again, isolated and refractory cases who responded to the anti-IL-6 receptor antibody after not responding to other biologics like Adalimumab, Infliximab or Abatacept, whose remission lasted for at least the follow-up months [65].

Tocilizumab seems effective for treating refractory uveitis-related cystoid macular edema [66]. However, one case of acute anterior uveitis with hypopyon and high IL-6 indices in aqueous humour was described 43 days after dropping off Tocilizumab, which was later resolved with corticoids. It has been postulated that as Tocilizumab does not inhibit the production of IL-6, but blocks its receptor, IL-6 production would be maintained and would still rise in fluids, which would explain the rebound inflammation after suspending therapy [67].

### 7.7. Interferon

Nowadays, numerous commercial IFN-α (α-2a, α-2b) and IFN-β (β-1a and β-1b) are available. The expression of hundreds of genes, including cytokine-codifying genes, growth factors, pro-apoptotic factors and adhesion molecules, among others, are stimulated by INFs. Therefore, INFs are able to integrate both innate and adaptive immune responses.

Kötter *et al.* [68] analyzed 36 clinical studies published about patients with refractory uveitis secondary to Beçhet and on Interferon treatment. These authors reported a partial or complete remission in 94% of these patients after 2–4 weeks. Both INF-α-2a and INF-β-1a have proven effective for treating uveitic cystoid macular edema in 70% of patients, but it tends to relapse when treatment stopped. Specifically, IFN α-2a therapy might act by causing Treg cells to recover their suppressive function to become beneficial thereof for suppressing intraocular inflammation and avoiding uveitis flare ups, which occur in patients with refractory Behçet uveitis [69]. Its use is not recommended in uveitis secondary to sarcoidosis because INF-induced sarcoidosis has been described as a side effect in some patients [70].

### 7.8. Rituximab

Rituximab is a monoclonal antibody against antigen CD20 on the surface of B-cells. It has been employed successfully to treat non-Hodgkin’s lynphoma, rheumatoid arthritis, systemic erythematous lupus, Wegener’s granulomatosis, and vasculitis. It is considered an alternative to refractory uveitis cases who do not respond to other drugs [71]. Therefore, Sadreddini *et al.* [72] reported a patient with Behçet and retinal vasculitis treated successfully with two doses of rituximab. These authors infused risuximab (1000 mg/dose), which the same dose was repeated two weeks after. Consequently, the patient remission was sustained for 24 months of follow-up [73]. Rituximab is also a good option to diffuse subretinal fibrosis uveitis syndrome, an unusual form of granulomatous multifocal choroiditis, described as widening areas of subretinal fibrosis which coalesce with subsequent macular involvement and visual loss [74].

### 7.9. Daclizumab

Daclizumab is a humanized mononuclear antibody of the IgG1 subtype that binds to the Tac epitope on the interleukin-2 (IL-2) receptor a-chain (CD25), and effectively blocks the formation of high-affinity IL-2 receptor. IL-2 increases the immune responses mediated by conventional T-cells, but Tregs also depend on IL-2. So IL-2 is critical for peripheral tolerance [75,76].

Wroblewski *et al.* [77] published a retrospective study which recruited 39 patients with refractory posterior uveitis who were treated with Daclizumab between 1997 and 2008. The mean treatment time was 40.3 months. In the patients on Daclizumab, the number of adjuvant immunosuppressors lowered from 1.89 to 1.17, and the number of periocular corticoids also lowered to 1.46. Thus we can deduce that Daclizumab is able to reduce concomitant treatment and to prevent outbreaks. Conversely, four patients developed solid malignant tumours after a mean time of 26 months since they started Daclizumab treatment.

In an acute animal model, Daclizumab diminished Th1 lymphocytes-related cytokines IL-2 and INF-γ by around 60%–70%, as well as protein concentration in aqueous humours, reduced uveal histopathological grading, and it also played a defensive role in endotoxin-induced oxidative stress [78].

### 7.10. Alemtuzumab

CD52 is a glycoprotein present in T and B lymphocytes, most monocytes, macrophages, NK cells and granulocytes. The antibody that inhibits CD-52 is known as Alemtuzumab, an IgG1k humanized antibody that binds to the CD-52 cell surface, which is indicated for chronic lymphatic leukaemia. According to Lockwood *et al.* [79], it seems somewhat capable of inducing remission in 72% of Behçet patients at six months, although relapses appeared in 38% of the patients after 25 months.

### 7.11. Others

Choroidal neovascularisation is a rare complication in uveitis, although it has been associated with punctate choroidopathy, multifocal choroiditis, serpiginous, and Vogt-Koyanagi-Harada. When antiVEGFs are present [80], such as Bevacizumab [81,82,83], Ranibizumab [84] or Aflibercept [85], they are good alternatives. Nevertheless, these antiVEGFs, which are usually injected intravitreally, may generate *per se* an immediate, transient, and mild eye inflammation like that demonstrated in animal models [86].

## 8. Conclusions

The use of biologics to treat uveitis has quickly extended in the past decade. Their achievement has underlined the key roles of inflammatory cytokines in the pathogenesis of inflammatory uveitis, principally TNF-α, IL-1, and IL-6. They have also refocused attention on T-cells and B-cells. Many patients with uveitis can benefit from a wide spectrum of biologics. These products are certainly useful when conventional immunosuppressor therapy fails or is not well tolerated, or also for treating concommitant ophthmic and systemic inflammation which could benefit from these medicines.

Nowadays, however, their use as a first-line therapy is not recommended in most patients, except in specific cases in which eyesight may be threatened, or in the other highly-specific cases mentioned herein.
